# Metagenomic next-generation sequencing for diagnosis of immune checkpoint inhibitor-associated pneumonitis: a retrospective comparative clinical performance study

**DOI:** 10.3389/fcimb.2026.1730022

**Published:** 2026-02-06

**Authors:** Ya-Lin Jiang, Shi-Zhen Dong, Yuan-Bo Xu, Jun-Li Fan, Yong-Mei Zhang, Shen-Shen Huang

**Affiliations:** 1 Department of Clinical Laboratory, The First Affiliated Hospital, and College of Clinical Medicine of Henan University of Science and Technology, Luoyang, China; 2 Department of Respiratory and Critical Care Medicine, The First Affiliated Hospital, and College of Clinical Medicine of Henan University of Science and Technology, Luoyang, China; 3 Department of Thoracic Oncology Surgery, The First Affiliated Hospital, and College of Clinical Medicine of Henan University of Science and Technology, Luoyang, China

**Keywords:** conventional microbiological testing, diagnostic value, immune checkpoint inhibitor-related pneumonitis, infectious pneumonia, mNGS

## Abstract

**Objective:**

To evaluate the diagnostic performance and clinical utility of metagenomic next-generation sequencing (mNGS) in distinguishing immune checkpoint inhibitor–related pneumonitis (CIP) from infectious pneumonia in cancer patients undergoing immunotherapy.

**Methods:**

A retrospective tertiary hospital cohort included 34 cancer patients (Feb 2022–Jan 2024) with prior ICI exposure, new/worsening respiratory symptoms, imaging infiltrates, and both mNGS and conventional microbiological testing (CMT). Final diagnoses were adjudicated by a multidisciplinary panel. We compared pathogen detection rates, sensitivity, specificity, and turnaround times (TAT) between mNGS and CMT.

**Results:**

In the infectious pneumonia group, mNGS detected pathogens in 17/18 cases (94%), whereas CMT detected only 6/18 (33%). In the CIP group, mNGS was negative in 14/16 cases (88%), compared with 11/16 negatives by CMT (69%). Using the adjudicated diagnosis as the reference, mNGS showed sensitivity 88%, and specificity 94%. In contrast, CMT’s sensitivity was 69%, and specificity 33%. The median TAT for mNGS was 24 hours (IQR 22–31 h), versus 121.5 hours (IQR 80.5–156 h) for CMT (P < 0.001).

**Conclusion:**

mNGS outperforms CMT in both diagnostic accuracy and timeliness for distinguishing CIP from infectious pneumonia among immunotherapy recipients. Incorporation of mNGS into the diagnostic workflow for suspected CIP may improve etiological discrimination and enable timely, individualized treatment. Further large-scale prospective studies are required to confirm these findings.

## 1 Introduction

Immune checkpoint inhibitors (ICIs), including antibodies targeting programmed death 1(PD-1), programmed death-Ligand 1(PD-L1), and cytotoxic T-lymphocyte-associated protein 4 (CTLA-4), have transformed the treatment landscape of multiple malignancies by unleashing antitumor immune responses. However, these therapies can precipitate immune-related adverse events (irAEs) due to unintended activation against self-tissues. Among these, immune checkpoint inhibitor–related pneumonitis (CIP) is one of the most serious pulmonary toxicities, often associated with considerable morbidity and mortality (Pozzessere et al., 2021).

The incidence of CIP varies across studies and populations. In meta-analyses of clinical trials, the incidence of any-grade pneumonitis with ICIs has been reported at ~3–5%, with higher rates of severe (grade ≥3) events observed for PD-1 inhibitors in comparison to PD-L1 inhibitors (e.g. 3.6% vs 1.3%) (Sternschein et al., 2018). Real-world cohorts frequently report higher incidence rates, possibly reflecting broader patient heterogeneity and comorbidities (Lin et al., 2024). The onset of CIP is variable, often within weeks to months after ICI initiation (range from ~9 days up to >19 months). Risk factors identified in multiple studies include preexisting lung disease (e.g. interstitial lung disease), prior thoracic radiotherapy, combination immunotherapy regimens, and baseline pulmonary inflammation markers (Zhou et al., 2025).

Crucially, there is currently no well-validated diagnostic approach that can reliably exclude infectious etiologies in patients with suspected CIP, despite the central importance of doing so for accurate diagnosis and appropriate management. Without such tools, clinicians must often rely on conventional microbiological testing (CMT), which has limited sensitivity and lengthy turnaround times, particularly for fastidious, rare, or coinfecting pathogens. This lack of validated exclusionary diagnostics contributes directly to diagnostic uncertainty, delays in correct therapy, and potential harm from either unnecessary immunosuppression or inappropriate antimicrobial use.

Clinically, CIP may present with nonspecific symptoms (cough, dyspnea, or low-grade fever) and radiographic features such as ground-glass opacities, consolidation, or interstitial abnormalities—patterns that substantially overlap with infectious pneumonia (Li et al., 2025a). Because of this overlap, CIP is often a diagnosis of exclusion, requiring systematic evaluation to rule out infection, tumor progression, radiation pneumonitis, or other interstitial lung diseases (Lin et al., 2024). Conventional microbiological testing—including cultures, multiplex PCR, antigen/serology tests, and standard panels—is routinely used to assess infectious etiologies in this context. However, such methods have several limitations in this setting: many fastidious, rare or coinfecting pathogens escape detection by targeted methods, limiting sensitivity and coverage; culture-based techniques often require days to weeks, delaying clinical decisions; patients already on empiric antimicrobials or with low pathogen burden may yield negative results; invasive sampling such as Bronchoscopy/BAL or lung biopsy can increase sensitivity, but are traumatic to patients (Lavalle et al., 2024; Xu et al., 2025b).

Given these drawbacks and the absence of validated diagnostic tools to reliably exclude infection in suspected CIP, clinicians frequently face uncertainty in distinguishing infectious pneumonia from CIP, leading to treatment dilemmas (e.g., starting steroids in unrecognized infection, or withholding immunosuppression for fear of infection).

Metagenomic next-generation sequencing (mNGS) is an emerging, unbiased approach that sequences all nucleic acids in clinical specimens (e.g. BALF, sputum, lung tissue), enabling simultaneous detection of bacteria, viruses, fungi, and parasites—without prior targeting. Several recent studies in immunocompromised patients and severe pneumonia cohorts have demonstrated that mNGS can substantially improve pathogen detection rates compared with conventional methods (Peng et al., 2021; Xu et al., 2023; You et al., 2024). For example, in a cohort of lower respiratory tract infections (LRTIs), mNGS identified pathogens in ~93.3% of cases versus ~55.6% by culture methods (Lai et al., 2025; Li et al., 2025c). Another recent study confirmed high positive rates of mNGS relative to culture/traditional PCR in both immunocompromised and immunocompetent patients with pulmonary infections (Li et al., 2025c). Furthermore, in immunocompromised ICU patients, mNGS has been shown to help refine antimicrobial regimens and inform targeted therapy (Zhao et al., 2024).

To address the critical gap in diagnostic tools for discriminating infectious pneumonia from CIP, we conducted a retrospective cohort study in cancer patients receiving ICIs who developed pulmonary infiltrates suggestive of either CIP or infectious pneumonia. Our objective was to compare the diagnostic performance (sensitivity, specificity, positive/negative predictive values), turnaround time, and incremental diagnostic yield of mNGS versus conventional microbiological testing in the discrimination of CIP from infectious pneumonia. We hypothesized that mNGS would outperform conventional methods in both accuracy and speed, thereby improving clinical decision-making in this challenging scenario.

## 2 Materials and methods

### 2.1 Study design

We conducted a retrospective cohort study at the First Affiliated Hospital of Henan University of Science and Technology, a tertiary referral center, covering the period from 1 February 2022 to 30 January 2024. The institutional ethics committee approved the protocol, and the need for informed consent was waived owing to the retrospective nature of the study (K-2025-B028).

**Figure 1:**
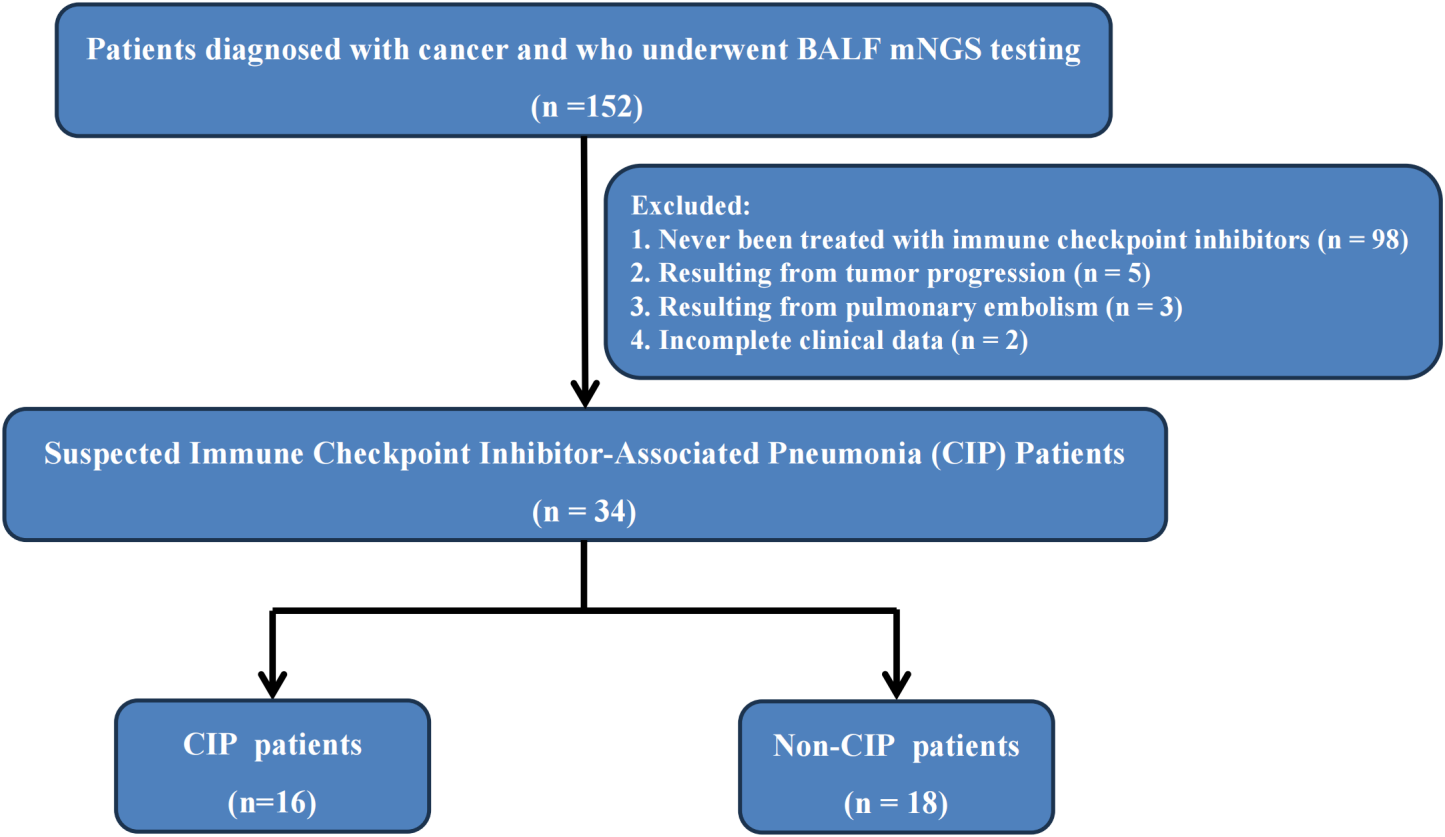
Flowchart.

This study enrolled cancer patients (solid tumors or lymphomas) who underwent at least one mNGS analysis on a respiratory sample during admission, and at least one form of conventional microbiological testing (CMT) for infection. Eligible participants were adults with histologically or cytologically confirmed malignancies who had received at least one dose of an immune checkpoint inhibitor (ICI; anti–PD-1, anti–PD-L1, or anti–CTLA-4). All patients were hospitalized for new or worsening respiratory symptoms, such as cough, dyspnea, or fever, accompanied by new pulmonary infiltrates on chest imaging that warranted etiologic evaluation for pneumonia or pneumonitis. To ensure comparability between diagnostic modalities, only patients who had undergone both mNGS testing on respiratory specimens during the index hospitalization and CMT as ordered by the treating physicians were included. Patients were excluded if they had malignancies not treated with ICIs, lacked CMT data, or had incomplete clinical information preventing definitive classification. After applying these criteria, a total of 34 patients were included in the final analysis. The flowchart of patient enrollment is illustrated in **Figure 1**.

All patients diagnosed with severe CAP receive empirical antimicrobial therapy according to the guidelines upon admission. Furthermore, comprehensive assessments including blood routine tests, procalcitonin (PCT), C-reactive protein (CRP) levels, microbiological analysis, microculture, and chest imaging examinations are promptly conducted. Treatment strategies are adjusted based on the results of microculture and mNGS.

### 2.2 Clinical data collection

Baseline clinical characteristics and outcomes were collected retrospectively for each enrolled patient upon admission. This included gender, age, presence of comorbidities (hypertension, diabetes mellitus, coronary artery disease, chronic obstructive pulmonary disease[COPD], cancer type, Type of ICI, etc.), laboratory parameters (including white blood cell count, neutrophil count, platelet count, CRP, PCT, etc.), length of hospital stay, duration of ICU stay, mechanical ventilation utilization, and In-hospital mortality. Laboratory results of all mNGS and CMT were recorded. CMT could include cultures (sputum, bronchoalveolar lavage [BAL] fluid, blood), nucleic acid PCR assays for respiratory pathogens, antigen tests, or serologies, as ordered by the treating physicians. We also recorded the time intervals from hospital admission to obtaining test results for mNGS and CMT. Treatments given (antibiotics, corticosteroids) and response were noted but are beyond the scope of this diagnostic analysis.

### 2.3 mNGS and conventional microbiology testing

All enrolled patients underwent bronchoscopy to collect bronchoalveolar lavage fluid (BALF), and the same BALF specimens were subjected in parallel to mNGS and CMT. For mNGS, in accordance with our prior published protocols (Huang et al., 2024): 1.2 mL BALF was mixed with 1.2 mL sterile buffer in 2 mL centrifuge tubes, followed by cell lysis using a BSP-100 oscillation disruptor (Hangzhou Jieyi Biotechnology), genomic DNA extraction with the MD013 kit (Hangzhou Jieyi Biotechnology), and library construction—including RNA reverse transcription for viral detection, enzymatic digestion, end repair, end adenylation, and adapter ligation—on the NGSmaster™ automated system (MAR002, Hangzhou Jieyi Biotechnology), with high-throughput sequencing conducted on an Illumina NextSeq 550 platform generating ~20 million 50-base pair single-end reads per library; for bioinformatic analysis and species identification (Huang et al., 2024), raw data were demultiplexed into fastq format, clean reads obtained by filtering short reads (<35 bp), low-quality reads (Phred score <20), and low-complexity sequences, human-origin reads removed via alignment to the GRCh38.p13 genome using Bowtie2 (Langmead et al., 2019), remaining microbial reads subjected to species identification with Kraken2 (Wood et al., 2019) and taxonomic assignments validated against a custom database of respiratory pathogenic microorganisms.

A microbe was reported as positive only if: (1) its read count exceeded a laboratory-validated threshold (established as the mean read count of negative control samples + 3 standard deviations) to exclude background noise; (2) no corresponding reads were detected in negative controls (sterile water and blank extraction controls); and (3) the relative abundance of the microbe accounted for ≥0.01% of total microbial reads (to rule out contamination). The mNGS turnaround time (TAT) was defined from the moment of BALF collection to dissemination of the final clinical report.

CMT encompassed standard-of-care assays on BALF, including bacterial, fungal, and mycobacterial cultures (with phenotypic identification via matrix-assisted laser desorption/ionization time-of-flight mass spectrometry [MALDI-TOF MS] and antimicrobial susceptibility testing using broth microdilution if growth occurred), Gram staining, acid-fast staining for mycobacteria, molecular PCR assays targeting common respiratory viruses (e.g., influenza A/B, respiratory syncytial virus, cytomegalovirus) and atypical pathogens (e.g., Mycoplasma pneumoniae, Chlamydia pneumoniae), and urinary antigen tests for Streptococcus pneumoniae and Legionella pneumophila serogroup 1. The CMT TAT was measured from BALF collection to the first positive result (for culture-positive cases) or—if all assays remained negative—to issuance of the final negative result report (after completion of 7-day culture incubation for bacteria/fungi).

### 2.4 Outcome definitions and diagnostic criteria

The primary outcome of this study was the final clinical diagnosis, classifying each patient as either CIP or infectious pneumonia. The final diagnosis was determined by the treating clinical team and confirmed by retrospective chart review, integrating all available clinical, radiologic, and laboratory data. The secondary outcomes focused on the comparative diagnostic performance of mNGS and CMT in distinguishing CIP from infectious pneumonia.

CIP was defined as a diagnosis of exclusion, based on a multidisciplinary assessment by pulmonology, oncology, and infectious disease specialists, in accordance with published literature and expert consensus guidelines (Chinese Thoracic Society CMA et al., 2025; Li et al., 2025a; Wang et al., 2022; Xu et al., 2025b). Eligible patients had a documented history of ICI therapy (anti–PD-1, anti–PD-L1, or anti–CTLA-4 antibodies), and developed new or worsening respiratory symptoms—including dyspnea, cough, fever, chest pain, or hypoxemia—accompanied by new pulmonary infiltrates on imaging such as ground-glass opacities, consolidations, or interstitial changes. Infectious etiologies (bacterial, viral, or fungal), tumor progression or infiltration, radiation pneumonitis, aspiration, and cardiogenic pulmonary edema were carefully excluded.

Infectious pneumonia was defined as pneumonia with clear clinical evidence of infection and microbiological confirmation of a causative pathogen.

To distinguish CIP from infectious pneumonia, all patients underwent comprehensive microbiologic evaluation, including both CMT and mNGS of BALF. The absence of any identifiable pathogen—particularly a negative mNGS result with sufficient sequencing depth—was considered strong supportive evidence against infection. All cases were independently reviewed by a multidisciplinary adjudication panel (pulmonology, oncology, infectious diseases) blinded to the study hypothesis. After integrating clinical, radiologic, microbiologic, and therapeutic response data, each case was classified by consensus as CIP or infectious pneumonia. Additionally, for cases with positive microbiological findings, the multidisciplinary adjudication panel further differentiated true infection from colonization by integrating predefined criteria that included pathogen load (quantitative mNGS read counts and relative abundance) and pathogen type, clinical presentation, inflammatory biomarkers, radiographic distribution patterns, and response to targeted therapy. Clinical improvement following ICI discontinuation and corticosteroid or immunosuppressive therapy, in the absence of response to antimicrobial treatment, was regarded as additional confirmatory evidence supporting the diagnosis of CIP.

### 2.5 Statistical analysis

Baseline characteristics were summarized for the overall cohort and compared between CIP vs infectious pneumonia groups. Continuous variables (e.g. age, CRP) were expressed as median with interquartile range (IQR) and compared using the Mann-Whitney U test. Categorical variables (e.g. sex, cancer type distribution) were compared using Chi-square or Fisher’s exact test as appropriate. The diagnostic performance of mNGS and CMT for identifying CIP was evaluated by calculating sensitivity, specificity, positive predictive value (PPV), negative predictive value (NPV), positive likelihood ratio (PLR), and negative likelihood ratio (NLR) for each method, using the final clinical diagnosis of CIP vs infectious pneumonia as the reference standard. We also constructed receiver operating characteristic (ROC) curves for each diagnostic modality. The ROC curve was generated by varying the stringency threshold for calling a positive pathogen (thus classifying CIP vs Infectious pneumonia). Finally, we compared the diagnostic turnaround times of mNGS vs CMT; since these were paired for each patient, a Wilcoxon signed-rank test was used to assess the difference in time to result. A two-tailed p-value < 0.05 was considered statistically significant. All analyses were performed using R (version 4.4.0).

## 3 Results

### 3.1 Bassline characteristics

This study is a retrospective cohort study that consecutively enrolled 152 patients diagnosed with cancer and who underwent BALF mNGS testing at the First Affiliated Hospital of Henan University of Science and Technology from February 2022 to January 2024. We excluded a total of 108 patients: 98 who had never been treated with immune checkpoint inhibitors, 5 whose pulmonary changes were judged to result from tumor progression, 3 who were found to have pulmonary embolism, and 2 whose clinical records were incomplete and thus unsuitable for proper adjudication. Ultimately, 34 patients were included in the study. Among these patients, 16 were adjudicated as CIP and 18 as infectious pneumonia. The patient enrollment process is illustrated in **Figure 1**.

Comparisons of demographic and clinical characteristics between the CIP and infectious pneumonia groups are summarized in **Table 1**. The median age was 64.5 years (IQR 58.0–70.0), with little difference between groups (CIP: 65.5 [58.0–70.5] vs infectious pneumonia: 63.5 [54.0–70.0], P = 0.400). Most patients were male (79.4%), and the proportion was higher in the CIP group (93.8% vs 66.7%, P = 0.090). There were no statistically significant differences in the distribution of comorbidities between groups, including hypertension, diabetes mellitus, COPD, and coronary artery disease (all P > 0.05). Smoking history was common (55.9%), with no significant difference (62.5% in CIP vs 50.0% in infectious pneumonia, P = 0.510). On laboratory testing, median WBC, neutrophil percentage, and platelet counts did not differ significantly. However, lymphocyte count was higher in the CIP group (1.18 ×10^9^/L vs 0.53 ×10^9^/L, P = 0.023), and PCT was lower (0.10 ng/mL vs 0.20 ng/mL, P = 0.045). C-reactive protein levels tended to be lower in CIP but did not reach statistical significance (55.31 vs 97.05, P = 0.073).

**Table 1:** Demographics and clinical characteristics of the study cohort.

Characteristics	Total (n=34)	CIP (n=16)	Infectious pneumonia (n=18) (n=18)	P values
Age, years	64.5 (58.0, 70.0)	65.5 (58.0, 70.5)	63.50 (54.0, 70.0)	0.400
Male, n(%)	27 (79.4)	15 (93.8)	12 (66.7)	0.090
Comorbidities				
Hypertension, n(%)	10 (20.9)	2 (12.5)	8 (44.4)	0.063
DM, n(%)	4 (11.7)	1 (6.3)	3 (16.7)	0.604
COPD, n(%)	3 (8.8)	2 (12.5)	1 (5.6)	0.591
CAD, n(%)	3 (8.8)	1 (6.3)	2 (11.1)	0.999
Smoking, n(%)	19 (55.9)	10 (62.5)	44 (50.0)	0.510
Laboratory detection				
WBC, x10^9/L	7.72 (5.28, 9.02)	7.98 (6.44, 9.27)	6.74 (4.54, 8.89)	0.200
NEU, %	77.0 (67.4, 86.1)	71.60 (63.35, 83.70)	83.10 (73.70, 87.10)	0.073
LYM, x10^9/L	0.88 (0.48, 1.37)	1.18 (0.77, 1.82)	0.53 (0.40, 1.16)	0.023
PLT, x10^9/L	205 (143, 255)	200 (152.5, 288.5)	219 (143, 255)	0.700
PCT, ng/ml	0.15 (0.09, 0.31)	0.10 (0.07, 0.16)	0.20 (0.11, 0.82)	0.045
CRP, ng/ml	79.81 (35.74, 123.52)	55.31 (23.30, 94.37)	97.05 (61.28, 124.71)	0.073
Cancer types				0.551
Lung cancer, n(%)	19 (55.9)	11 (68.8)	8 (44.4)	
Gastric Cancer, n(%)	1 (2.9)	1 (6.3)	0 (0.0)	
Cardiac Cancer, n(%)	3 (8.8)	1 (6.3)	2 (11.1)	
Renal Cancer, n(%)	1 (2.9)	0 (0.0)	1 (5.6)	
Esophageal Cancer, n(%)	5 (14.7)	3 (18.8)	2 (11.1)	
Lymphoma, n(%)	1 (2.9)	0 (0.0)	3 (16.7)	
Liver Cancer, n(%)	1 (2.9)	0 (0.0)	1 (5.6)	
Breast Cancer, n(%)	1 (2.9)	0 (0.0)	1 (5.6)	
Type of ICIs				0.405
anti–PD-1, n(%)	26 (76.5)	13 (81.3)	13 (72.2)	
anti–PD-L1, n(%)	7 (20.6)	2 (12.5)	5 (27.7)	
anti–CTLA-4, n(%)	1 (2.9)	1(6.3)	0 (0.0)	
LOS, days	21.5 (15.0, 33.0)	21.0 (16.5, 29.5)	23.5 (15.0, 42.0)	0.800
ICU admission rate, n (%)	13 (38.2)	4 (25.0)	9 (50.0)	0.253
Mechanical ventilation utilization rate, n (%)	8 (23.5)	2 (12.5)	6 (33.3)	0.233
In-hospital mortality rate, n (%)	7 (20.6)	1 (6.3)	6 (33.3)	0.090

CIP, Immune checkpoint inhibitor-associated pneumonitis; DM, diabetes mellitus; COPD, chronic obstructive pulmonary disease; CAD, coronary artery disease; ICI, immune checkpoint inhibitor; WBC, white blood cell count; NEU, neutrophilic granulocyte percent; LYM, lymphocyte count; LYM%, lymphocyte percentage; PLT, platelet count; PCT, procalcitonin; CRP, C-reactive protein. LOS, length of stay.

Regarding cancer types, lung cancer was the most common (55.9%), followed by esophageal cancer. The distributions of tumor types across groups were not significantly different (P = 0.551). For immune checkpoint inhibitor types, anti–PD-1 was most frequently used (76.5% overall), followed by anti–PD-L1 (20.6%) and anti–CTLA-4 (2.9%), with no significant between-group difference (P = 0.405). Hospital course and outcomes were broadly comparable: median length of stay was 21.5 days (IQR 15.0–33.0), ICU admission occurred in 38.2%, and mechanical ventilation in 23.5%. These outcomes did not differ significantly between the CIP and infectious pneumonia groups (all P > 0.05). In-hospital mortality was higher in the infectious pneumonia group than in the CIP group (33.3% vs. 6.3%), although this difference did not reach statistical significance (P = 0.090).

### 3.2 Pathogen detection by mNGS vs CMT

Among the 18 patients with infectious pneumonia, mNGS successfully detected at least one causative pathogen in 17 cases (94%). The identified pathogens included common bacteria (e.g., Streptococcus pneumoniae, Staphylococcus aureus) and opportunistic organisms (e.g., Pneumocystis jirovecii, Nocardia species, Aspergillus species). In contrast, CMT detected pathogens in only 6 of the 18 infection cases (33%). Conversely, among the 16 patients ultimately diagnosed with CIP, mNGS correctly yielded negative results (no significant pathogen detected) in 14 cases (88%), while CMT was negative in 11 of 16 cases (69%). The two false-positive mNGS results in CIP patients involved Pseudomonas aeruginosa and Aspergillus species. The former was likely due to colonization in patients with chronic lung disease, whereas the latter was considered attributable to concomitant aspergillosis rather than immune-related pneumonitis.

Overall, the pathogen detection rate of mNGS was 56% (19 out of 34 patients had at least one pathogen identified), which was significantly higher than the 32% yield achieved by conventional microbiological testing.

### 3.3 Diagnostic performance of mNGS and CMT

We further analyzed the diagnostic performance of mNGS and CMT to distinguish CIP from infectious pneumonia, especially infectious pneumonitis. Using the final clinical diagnosis as the gold standard, mNGS significantly outperformed CMT in distinguishing CIP from infectious pneumonia. mNGS achieved a sensitivity of 88% and specificity of 94%, with a PPV of 93% and NPV of 89%, and favorable likelihood ratios (PLR 15.75, NLR 0.13). In contrast, CMT’s sensitivity and specificity were only 69% and 33%, respectively, with a PPV of 48%, NPV of 55%, PLR of 1.03, and NLR of 0.94 (**Table 2**). These results show that mNGS provides a robust diagnostic signal, reliably ruling in or out CIP, while conventional testing alone offers limited utility in this context.

**Table 2:** Comparison of diagnostic performance indicators between mNGS and CMT.

Metric	mNGS estimate (95% CI)	CMT estimate (95% CI)
Sensitivity	88% (62%–98%)	69% (41%–89%)
Specificity	94% (73%–100%)	33% (13%–59%)
PPV	93% (68%–100%)	48% (27%–69%)
NPV	89% (67%–99%)	55% (23%–83%)
PLR	15.75 (2.32–106.76)	1.03 (0.65–1.64)
NLR	0.13 (0.04–0.49)	0.94 (0.35–2.49)

CMT, Conventional microbiological testing; CI, Confidence interval; mNGS, Metagenomic next-generation sequencing; NLR, Negative likelihood ratio; NPV, Negative predictive value; PLR, Positive likelihood ratio; PPV, Positive predictive value.

### 3.4 Diagnostic turnaround time of mNGS and CMT

**Figure 2:**
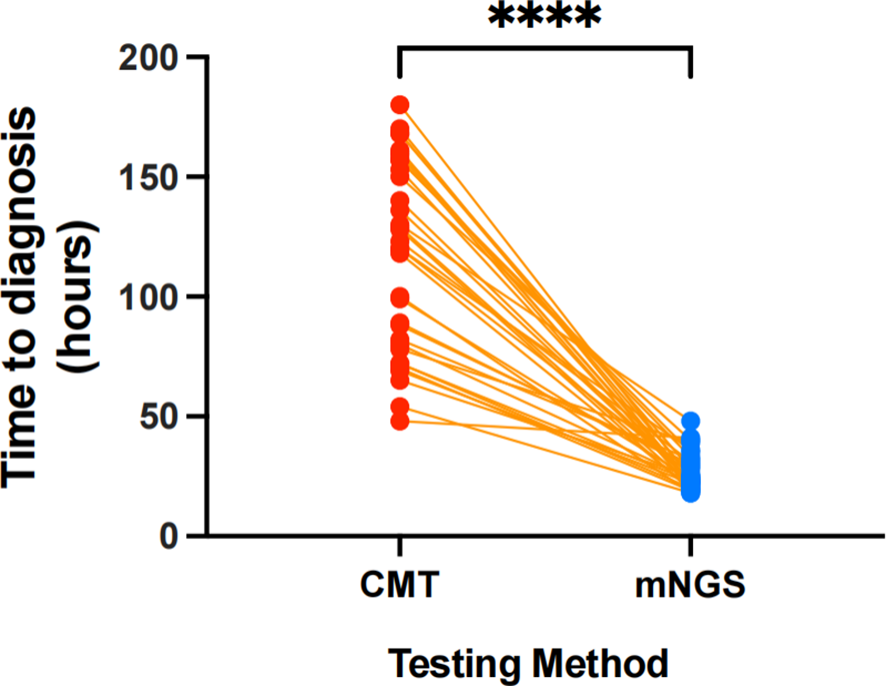
Comparison of time to diagnosis (in hours) between CMT and mNGS. Each paired dot connected by an orange line represents an individual sample tested by both methods. Red dots denote time to diagnosis with CMT, and the blue box-and-whisker plot represents time to diagnosis with mNGS. The **** indicates a highly significant difference (P < 0.0001) in time to diagnosis between the two methods, with mNGS demonstrating a substantially shorter time to diagnosis compared to CMT.

An important practical finding was the difference in time to obtain results. The time from sample collection to diagnostic result was significantly shorter with mNGS compared to conventional methods. The median turnaround time for mNGS was 24 hours (IQR 22.0–30.8 hours), whereas for the composite of conventional tests it was 121.5 hours (approximately 5 days; IQR 80.5–156 hours). This difference was highly statistically significant (P < 0.001) (**Figure 2**). In our study, the substantially faster turnaround of mNGS meant that, for many patients, we could identify an infectious organism or conclude its absence well before the conventional tests were finalized.

## 4 Discussion

In this retrospective cohort of cancer patients receiving ICIs with suspected pneumonitis, we found that mNGS markedly outperformed conventional microbiological testing in discriminating CIP from infectious pneumonia: its sensitivity reached 88% and specificity 94%, far exceeding the 69% and 33% achieved by traditional methods. To our knowledge, this is among the first studies to directly compare mNGS and standard diagnostics in the context of ICI-related pneumonitis. These findings highlight two crucial advantages of mNGS in this setting: superior diagnostic accuracy and significantly shorter time to etiologic determination. Importantly, mNGS’s ability to more confidently exclude infection addresses a core clinical challenge in CIP—namely, that CIP diagnosis often requires robust exclusion of infectious etiologies as a prerequisite for safe initiation of immunosuppression, an issue repeatedly emphasized in CIP diagnostic consensus and expert reviews.

Although numerous prior investigations have documented the added value of mNGS in improving pathogen detection in immunocompromised or critically ill patients, most focused on general pneumonia cohorts rather than the specific challenge of differentiating CIP. For example, in immunocompromised hosts, BALF mNGS has been shown to boost microbial detection rates by 20~40 percentage points over conventional tests, particularly in diagnosing opportunistic infections such as Pneumocystis jirovecii (with sensitivity improvements from ~28% to ~100%) (Xin et al., 2025). However, these studies did not address the competing diagnostic scenario where absence of pathogen detection itself carries diagnostic weight in favor of noninfectious immune-mediated lung injury—a central requirement in CIP diagnostic pathways highlighted by recent research.

By contrast, the literature on CIP strongly emphasizes the clinical and radiographic overlap between immune-mediated pneumonitis and infectious pneumonia and warns of frequent misclassification, which can lead to either overt immunosuppression in occult infection or delayed steroid therapy in true CIP (Li et al., 2025a). Prior reviews and expert consensus acknowledge that CIP diagnosis often hinges on exclusion of infectious etiologies, yet effective tools to aid that exclusion remain limited (Wang et al., 2022; Xu et al., 2025b). In other words, while mNGS has been advocated for infection diagnosis, its direct role in ruling out infection to support CIP diagnosis has been relatively unexplored in existing studies.

Our study helps fill this gap by demonstrating that in a cancer population treated with ICIs and presenting with suspected lung injury, mNGS not only detects pathogens when present but also yields negative results with high confidence, thereby offering strong support for CIP in appropriate clinical contexts. In our cohort of cancer patients treated with ICIs who developed suspected lung injury, mNGS markedly outperformed conventional microbiological methods in diagnostic accuracy. With a sensitivity of 88% and specificity of 94% (versus 69% and 33% for CMT), mNGS is capable not only of reliably identifying true CIP cases (by “excluding” pathogens in immune-related pneumonitis) but also of detecting infection when present. Practically, a negative mNGS result (no pathogen detected) carried a positive predictive value for CIP of ~93%, which is of critical importance: misdiagnosing CIP in a patient with occult infection and proceeding with immunosuppression could have serious consequences. At the same time, mNGS’s high specificity (94%) means that it seldom misses infection—in our series, only one patient with a real infection had a false-negative mNGS, whereas conventional testing missed 12 infections. Conversely, a positive mNGS result (pathogen identified) effectively ruled out CIP in nearly all cases (negative predictive value ~89%), alerting clinicians to an underlying infectious etiology. Conventional methods, with their NPV of ~55%, left much greater ambiguity: negative cultures or antigen tests could not reliably suggest noninfectious pneumonitis, because over half of true infections in our series would have been missed by such tests. This supports a clinical decision-making pathway where early mNGS results can meaningfully shorten the period of diagnostic uncertainty and guide whether to initiate immunosuppressive therapy or targeted antimicrobial treatment.

These findings underscore that, in an ICI-treated cancer population with suspected lung injury, mNGS not only enhances pathogen detection when infection is present, but more crucially offers a high-confidence “no pathogen” result to support a diagnosis of CIP in the appropriate clinical setting. This superior performance is especially relevant in light of the historically low yields of conventional microbiological methods (e.g. culture-based detection rates often reported around 30–35% in immunocompromised patients), and in our series, CMT detected pathogens in only ~32% of cases—leaving many infections undiagnosed by traditional approaches (Lu et al., 2022; Miao et al., 2018).

Another major advantage of mNGS observed in our study was its substantially shorter turnaround time. The median interval from sample collection to result was 24 hours (IQR 22–30 h), compared with approximately 121 hours (IQR 80–156 h) for conventional microbiological testing—nearly a fivefold difference. This acceleration is consistent with prior reports in immunocompromised pneumonia, where mNGS reduced time-to-diagnosis from 4–6 days (for culture or PCR workflows) to roughly 1 day (Li et al., 2025b).

This difference carries profound clinical importance. Patients with CIP may deteriorate rapidly and often require timely initiation of corticosteroid therapy to control immune-mediated inflammation. Yet clinicians typically hesitate to start high-dose steroids until infection has been confidently excluded. With traditional diagnostics taking nearly a week, this creates a prolonged period of uncertainty in which broad-spectrum antimicrobials are empirically prescribed, or steroids are delayed—both of which can worsen outcomes. In current practice, this often leads to a combined empiric treatment strategy that includes both high-dose steroids and broad-spectrum antibiotics, reflecting the difficulty in distinguishing immune-mediated lung injury from infection based on clinical and radiographic features alone (Tiu et al., 2022). Faster and more accurate mNGS diagnostics have the potential to shift this paradigm. A rapid negative mNGS result—available within ~24–48 hours in our cohort—can provide clinicians with greater confidence in excluding infection, supporting earlier and more targeted use of corticosteroids while reducing unnecessary antibiotic exposure. Conversely, early identification of a pathogen by mNGS can justify prioritizing pathogen-directed antimicrobial therapy and withholding or de-escalating immunosuppression to avoid exacerbating an underlying infection. For instance, in two of our cases—one involving a COPD patient with CIP complicated by Pseudomonas aeruginosa pneumonia and another with CIP plus Aspergillus infection—mNGS rapidly detected the respective pathogens, prompting prompt initiation of targeted antimicrobials; however, standalone anti-infective therapy failed to fully alleviate respiratory deterioration, as immune-mediated lung injury from CIP persisted. This necessitated the tailored addition of glucocorticoids to control inflammation, leading to significant clinical improvement.

In our cohort, for example, initiation of corticosteroids was delayed in several patients until pathogen exclusion by conventional methods—a process that often took multiple days—leading to prolonged empiric antibiotic use. By contrast, in patients where mNGS results were available early, clinicians were able to tailor therapy more rapidly: early negative results supported timely steroid initiation, and positive mNGS findings prompted focused antimicrobial therapy and avoidance of unnecessary immunosuppression. This represents a clinically actionable decision pathway, where mNGS results directly informed the balance between anti-inflammatory and anti-infective strategies, potentially reducing the risks associated with both overt immunosuppression and unnecessary antibiotic usage.

Quantitatively, this acceleration parallels recent studies in critically ill and immunosuppressed populations, where mNGS shortened diagnostic turnaround by 72–80 hours on average and improved time-to-appropriate therapy by 2–3 days (Li et al., 2025b) (Sears et al., 2019). Such gains may be especially impactful for CIP, where earlier diagnostic clarity directly informs whether to escalate immunosuppression or intensify antimicrobial coverage.

In our cohort, mNGS identified a range of bacterial, fungal, and viral pathogens that are clinically relevant and similar to those encountered in other infectious respiratory diseases. Detected organisms included Streptococcus pneumoniae, Nocardia spp., Aspergillus spp. (including Aspergillus), Pneumocystis jirovecii, Staphylococcus aureus, SARS-CoV-2, Pseudomonas aeruginosa, and Haemophilus influenzae. These taxa span common bacterial, fungal, and viral agents that contribute to lower respiratory tract infections in both immunocompromised and immunocompetent populations, reflecting the broad detection capability of mNGS in diverse infectious settings (Diao et al., 2022).

Despite its advantages, mNGS faces key limitations that merit careful consideration. First, cost and accessibility remain barriers in many centers, though prices are trending downward as sequencing becomes more common. Second, interpretation of positive findings is complex: mNGS may detect colonizers, contaminants, or latent organisms rather than true pathogens, particularly in low-microbial burden samples. Distinguishing clinically relevant signals often requires correlating read counts, abundance metrics, and clinical context. In a recent review, Simner et al. highlight that detection of microbial DNA does not always equate to infection, due to background laboratory or reagent contamination (Simner et al., 2018). Xu et al. similarly emphasize that positive mNGS reports may include incidental organisms requiring expert adjudication. In our study, two CIP patients had mNGS-detected species ultimately judged incidental (Xu et al., 2025a). Overall, the false-positive rate was low, but expert interpretation—often with infectious disease consultation—is essential to avoid misattribution.

There are also limitations in our study. First, our cohort included only 34 patients, reflecting the rarity of CIP and the single-center design; such a modest sample size may reduce the precision and generalizability of our sensitivity and specificity estimates, despite the observed effect sizes being large. Second, the retrospective design introduces inherent selection bias. For instance, mNGS may have been more often ordered in atypical or more severe cases, and relies on the completeness and accuracy of medical records in determining the reference diagnosis. We attempted to mitigate this by applying rigorous diagnostic criteria and employing blinded, multidisciplinary adjudication, but residual misclassification bias cannot be excluded. Third, although we compared the diagnostic performance of mNGS with CMT, the heterogeneity of CMT methods and the limited number of positive culture-based results precluded meaningful threshold variation and robust ROC-based analyses, restricting more granular comparisons across diagnostic modalities. Finally, although we measured diagnostic turnaround times, we did not formally evaluate downstream effects on clinical outcomes (e.g. time to appropriate therapy, duration of hospital stay, or mortality), limiting inferences about the ultimate patient benefit of mNGS. Future prospective, multicenter studies with larger sample sizes should investigate whether mNGS-guided diagnosis yields tangible improvements in clinical endpoints (shorter hospitalization, improved survival, cost-effectiveness), and validate standardized reporting thresholds and interpretation frameworks to optimize its use in CIP management.

In conclusion, our study highlights the effectiveness of mNGS as a diagnostic tool for suspected ICI−related pneumonitis. Relative to conventional microbiological testing, mNGS demonstrated substantially greater accuracy in differentiating CIP from infectious pneumonia and provided results in a fraction of the time, potentially enabling earlier, evidence−based therapeutic decisions. In patients receiving immunotherapy who present with pulmonary symptoms, early application of mNGS on BALF or other respiratory specimens may help rapidly exclude or confirm infection, thereby guiding optimal use of immunosuppression and antimicrobial therapy. However, this study’s retrospective design and single−center cohort with a modest sample size limit the generalizability of our findings, and further prospective, multicenter research with economic evaluation is needed to validate these results and clarify clinical impact. Taken tosgether, our experience suggests that mNGS can meaningfully improve both the precision and timeliness of diagnosis in this challenging clinical scenario, supporting its integration into diagnostic protocols for CIP with appropriate contextual interpretation.

## Data Availability

The data that support the findings of this study have been deposited into CNGB Sequence Archive (CNSA) of China National GeneBank DataBase (CNGBdb) with accession number CNP0005479.
